# Examining Menstrual Tracking to Inform the Design of Personal Informatics Tools

**DOI:** 10.1145/3025453.3025635

**Published:** 2017-05-02

**Authors:** Daniel A. Epstein, Nicole B. Lee, Jennifer H. Kang, Elena Agapie, Jessica Schroeder, Laura R. Pina, James Fogarty, Julie A. Kientz, Sean A. Munson

**Affiliations:** DUB Group, University of Washington

**Keywords:** Menstrual tracking, menstrual cycle, period, personal informatics, lived informatics, women's health, inclusivity

## Abstract

We consider why and how women track their menstrual cycles, examining their experiences to uncover design opportunities and extend the field's understanding of personal informatics tools. To understand menstrual cycle tracking practices, we collected and analyzed data from three sources: 2,000 reviews of popular menstrual tracking apps, a survey of 687 people, and follow-up interviews with 12 survey respondents. We find that women track their menstrual cycle for varied reasons that include remembering and predicting their period as well as informing conversations with healthcare providers. Participants described six methods of tracking their menstrual cycles, including use of technology, awareness of their premenstrual physiological states, and simply remembering. Although women find apps and calendars helpful, these methods are ineffective when predictions of future menstrual cycles are inaccurate. Designs can create feelings of exclusion for gender and sexual minorities. Existing apps also generally fail to consider life stages that women experience, including young adulthood, pregnancy, and menopause. Our findings encourage expanding the field's conceptions of personal informatics.

## Introduction

Personal tracking for self-knowledge is commonplace, from recording finances for accountability to tracking location for pure curiosity. Health tracking has perhaps captured the most attention, with nearly 70% of US adults tracking a health indicator [14]. However, relatively little attention has been paid to tracking factors specific to women's health^1^, including where a woman is in her menstrual cycle. When Apple HealthKit launched in 2014 without support for menstrual data, the public was outraged over the exclusion of such an essential aspect of health tracking [11]. Apple later added this feature, but its exclusion sparked a conversation about inclusivity in design of personal tracking tools [33].

We consider menstrual tracking through the lens of personal informatics, with two goals. We first contribute to an ongoing conversation on women's health in HCI (e.g., [1]) by examining the practice of menstrual cycle tracking. We offer an understanding of why and how women track their menstrual cycles, focusing on how they use technology to do so. Second, we identify design challenges and concerns in digital tools for menstrual cycle tracking, drawing upon such insights to offer guidance and challenge current broader assumptions in the design of personal informatics tools.

Although not about tracking a behavior, menstrual cycle tracking fits Li et al.'s definition of personal informatics as tracking to obtain self-knowledge [25]. The practice of menstrual cycle tracking challenges many assumptions of personal informatics. For example, women often track their menstrual cycles without an explicit goal of action, but instead for awareness of their place in their menstrual cycle. Understanding the differences and commonalities between menstrual cycle tracking and other domains of personal informatics extends how we as a field consider personal informatics and design our personal informatics tools.

Toward these goals, we collected and analyzed data from three sources. We first collected and coded 2,000 reviews of popular menstrual tracking apps on the iPhone App Store and Android Market. We then surveyed 687 people to understand their practices around tracking menstrual cycles. We finally conducted follow-up interviews with 12 survey respondents to gather in-depth perspectives of those practices.

In this paper we contribute:

- An empirical description of *why* women track their menstrual cycles. Women track to better understand their bodies and mental states, to have materials prepared for their period, to predict ovulation, and/or to describe their menstrual cycle to their doctors. These practices differ from the traditional personal informatics focus on tracking one's behavior, rather than one's experiences.
- An understanding of *how* women track their menstrual cycles. We identify six common methods: using dedicated apps, recording in digital calendars, using paper calendars or diaries, following birth control intakes or schedules, noticing early bodily symptoms, and simply remembering.
- A discussion of *problems and issues* associated with menstrual cycle tracking. We specifically consider concerns about the indiscrete nature of tracking, inclusivity, and varied and evolving use cases for the same tool.

We then discuss the broader implications of our findings for personal informatics, considering how the design of personal informatics tools can be informed by problems and issues women commonly encounter in menstrual cycle tracking.

## Background

The study of menstrual cycle tracking builds on prior research in technology for women's health and personal tracking.

### Women's Health and HCI

The HCI community has a rich history of studying technology in support of women at different stages of pregnancy and motherhood. Women often turn to web searches, apps, and social media during pregnancy and parenting for information on whether their experience is normal [13,23,28]. Many turn to social networks for support, information, and often commiseration [28,29,37]. HCI has further considered designs for technology supporting pregnancy and motherhood, including rethinking the experience of breastfeeding [4,10] and aiding in tracking child development [22,39]. Peyton et al. describe a *pregnancy ecology* to aid in design, shifting from a focus on a woman's activity, diet, and weight tracking to supporting her information seeking, self-knowledge, and social needs [34].

Prior research often focuses on maternal health. Designing and understanding technology for broader women's health has received relatively limited attention [1]. One design in the space is Labella, underwear with a visual marker and an app designed to help women explore their vaginal and pelvic region with a goal of breaking social taboos and promoting exploration and self-understanding [2]. Another is Help Pinky, a digital game aimed to bridge knowledge about menstruation and puberty in a rural Indian community with limited support and knowledge [18]. Work by Stawarz et al. is most closely related to menstrual cycle tracking [38]. They suggest that current pill reminder apps, including birth control apps, fail to effectively address forgetfulness, and that apps do not always integrate into people's varied routines.

Many design explorations of women's health technology have taken a feminist HCI approach [2,10], which stresses engaging with the perspectives of marginalized groups that are typically left out of the design process. Our work makes what Bardzell defines as a *critique-based* contribution [5] by “*analyz[ing] designs… to expose their unintended consequences*”, such as the downsides to normative design choices. In our work, we highlight ways in which tracking apps fail to support the marginalized populations of gender and sexual minorities. We adopt many aspects of Bardzell and Bardzell's Feminist HCI methodology [6], such as indicating our goals as technologists in our interactions with participants.

However, our research team's expertise is in personal informatics technology, rather than feminist theory. We therefore primarily approach this study through the lens of personal informatics, offering design insights for future tools. Although we do not connect our findings to feminist theory, we acknowledge the opportunity for considering personal informatics design through a feminist lens.

### Personal Informatics in Everyday Life

Li et al. define personal informatics as self-knowledge gained by collecting and reflecting on personal data [25]. Early models of personal informatics describe how people use technologies to collect and integrate data toward then acting on the collected data, typically with a goal of behavior change. However, personal tracking technology is now a part of people's everyday lives and is not necessarily associated with a self-improvement goal [35]. People track for other motivations, including pure curiosity and a desire to instrument a particular activity [12]. In this paper, we consider the everyday life experiences of menstrual cycle tracking to understand people's varied motivations, uses, and goals.

Personal informatics research has often investigated health and wellness by developing or studying digital tools to help people track and understand personal data. However, people also use other means to track their health. A 2013 Pew survey notes that 44% of US adults who track a health factor do so only in their heads, with 34% tracking on paper [14]. Digital systems for personal tracking offer benefits over paper systems, including improving accuracy and ease of entry while maintaining usability [43]. However, digital tools still sometimes fail to support people's needs or goals, and tracking on paper is still prolific. As shown in research on personal financial tracking, paper systems people use for personal tracking may or may not resemble digital methods [21]. People appreciate the flexibility paper affords, more than a technical solution focused on data aggregation. Women similarly use a variety of methods to track their menstrual cycles, from digital to memory-based.

A few studies have explored personal tracking in the context of sex and pregnancy. Lupton highlights self-quantifying components of apps for tracking sex performance (e.g., stamina, number of partners) as well as fertility indicators (e.g., ovulation, body temperature) [27]. Apps for tracking pregnancy often offer information on how the fetus should be developing and provide reassurance and advice about parenting [28]. Many apps for tracking pregnancy focus on associated risks, while others highlight associated joys [41]. However, little research focuses on examining the needs and challenges of menstrual cycle tracking.

## Data Collection and Analysis

We gathered data from three sources: online app reviews, a survey of women's tracking practices, and follow-up interviews with some survey participants. Table 1 summarizes participant demographics. The supplemental materials contain more detailed participant demographics, our analysis codebooks, as well as survey and interview materials including consent descriptions. We quote app reviews with identifiers AXXXX, survey responses with SXXX, and interviews with IXX.

### Method 1: App Reviews

In January 2016, we searched for “period tracker” on the Android Market and Apple App Store and selected the 12 most reviewed apps (9 distinct apps, as 3 were cross-platform). We downloaded the 2,000 most recent app reviews (120-200 per app, with variability caused by rate limits in our download script). We wanted to determine what characteristics of specific apps people like or dislike, rather than general opinions of the apps themselves. We therefore focused on open-ended review text, ignoring review scores.

We analyzed review data through a grounded approach. Two researchers open coded the reviews before paring down to 6 codes most relevant to our research questions. Another then coded the entire corpus per these 6 codes, with two additional researchers coding 25% each (κ=0.66-0.80). One of the researchers who defined the codes broke any ties.

### Method 2: Survey

After analyzing the app reviews, we designed a survey to address open questions about why and how women track. To understand whether menstrual tracking practices differ in a generation who grew up with apps available or if practices differ based on experience with menstruation, we developed the survey to reach both teenagers (13-18) and adults (18+). The survey first asked people whether or how they monitor their menstrual cycle. For those who currently or previously tracked, we asked primarily open-ended questions to understand how people track their menstrual cycle and what they like and dislike about their method.

We obtained IRB approval for this study from our university as minimal risk research with a waiver of parental consent, because requiring parental consent would impact the ability to conduct the research and could increase risk of participation (e.g., leading parents to make inferences about a minor's behavior). Adults granted consent after reading a description of the study. Minors assented to participation after reading a similar description adjusted for a grade school reading level.

We recruited 690 survey respondents via posts to Facebook, Twitter, and a Reddit subreddit targeted at teenagers. These participants were entered into a drawing for a $100 gift card to Amazon or Starbucks. We excluded 3 spam responses from our analysis (free text such as “*yes*” and “*I like*” for questions asking for an explanation), leaving 687 responses.

Three researchers first read the open-ended survey responses and discussed potential codes. The first author open coded the responses before condensing to 14 codes most relevant to our research questions. Two researchers then coded 10% of the data. Code agreement varied on this initial pass (κ=0.31-1, with 0.80 or higher for 10 codes). The two researchers arbitrated the disagreements until reaching 100% agreement, and one researcher then coded the remainder of the data.

### Method 3: Interviews

Our analysis of survey responses revealed areas we wanted to explore in more detail, including situations where tracking was uncomfortable and how menstrual tracking data is discussed with healthcare providers. In sampling for the interviews, we contacted 19 survey respondents who had agreed to be contacted for an interview. We aimed for diversity in experiences and backgrounds (including race, gender, sexual minorities, and health conditions) rather than representativeness. 12 responded to our request. Two researchers conducted each interview via phone or Skype, with one leading the interview and the other taking notes and asking follow-up questions. Interviews lasted about 30 minutes, and we compensated each participant with a $20 gift card to Amazon or Starbucks. An external service transcribed the interviews.

The researchers discussed major themes and identified 10 codes. Each transcript was then coded once by a researcher who did not participate in that interview, which helped each researcher become familiar with more interview data. We did not conduct inter-rater reliability on the interview data. It is rarely calculated on semi-structured interview data because people can apply the same code to different parts of a conversation [3]. The interviews were conducted under the same minimal risk IRB as the survey.

### Limitations

As HCI researchers, we designed our study to inform the design of menstrual cycle tracking technology through the lens of personal informatics. Our research methods were designed with a bias toward understanding how women currently use technology to track. Women often track their menstrual cycle through non-technological methods or in their heads, and some do not formally track at all. The prevalence of different tracking methods we report should not be interpreted as representative of any particular population, as our results likely exaggerate technology use.

The demographic makeup was heavily influenced by the research team's social networks and the Reddit population. Both participant groups likely skew WEIRD: Western, Educated, Industrialized, Rich, and Democratic [16]. Most participants were from the United States. Compared to the US population [17], a higher percentage of survey respondents identified their race as “White” or “Asian”, while fewer identified their race as “Black or African American” or “Hispanic or Latino.” We did not specifically target our recruitment at this population.

Although we focus on broad implications for menstrual tracking, substantial care must be taken when applying our findings to understanding tracking by women in cultures and ethnic groups underrepresented in our study. Future work is necessary to understand the challenges, motivations, and methods of tracking of women from other cultures, education levels, and economic statuses. As one example, S255 identifies as an Orthodox Jew, and tracks her cycle by “*remember[ing] what day I came back from the mikveh last*,” a purification ritual which immediately follows menstruation.

The keywords we selected when searching for apps led us to examine apps focused on period tracking rather than fertility and pregnancy. Many apps specifically aim to help women track factors important to becoming pregnant. We did not specifically investigate how women use these apps, as we were interested in the lived experience of menstrual cycle tracking. However, many apps that are described or marketed as period trackers prominently include fertility and pregnancy features. This point is discussed in our results.

## Why Women Track Menstrual Cycles

We surfaced five reasons women track their menstrual cycles. Women track to: (1) be aware of how their body is doing, (2) understand their body's reactions to different phases of their cycle, (3) be prepared, (4) become pregnant, and (5) inform conversations with healthcare providers. Participants were typically motivated by multiple factors.

Many women in our study viewed menstrual tracking as a “*general health check*” (S92). S250 describes tracking as an indication that her body is doing well: “*to be aware of my body, making sure it's happy and healthy*.” A232 agrees, noting “*it's a huge help in really getting to know your body*.” These people quickly glance at the data for awareness of their health, similar to idea of a “financial touch” that many people work to gain from tracking personal finances [21].

Survey respondents often tracked their menstrual cycles to understand their body's physical and emotional reactions at different phases of their menstrual cycle, and to verify and predict their body's response. S466 tracks to “*guess if cramping is likely to get worse*”, while S73 tracks “*so I know I'm not crazy when I start to PMS and so I can up my dosage of my anxiety meds*.” The tracked data can also be used to make sense of current experiences. S465 notes “*sometimes I'm really emotional and irrational and I can look at my tracker, see that my period is due in a week or less and chill out and realize I'm PMSing instead of having real feelings*.”

Participants reported tracking to be prepared, “*so it doesn't surprise me*” (S27). They often did so to ensure necessary materials are accessible, such as “*to predict when I will need pads and tampons*” (S135). The tracked menstrual cycle data informs decisions in women's everyday lives, echoing the lived experience of tracking [35]. People plan life events around their cycle, from leisure activities: “*I can literally plan my vacations and excursions around my time*” (A1122), to intimacy: “*I also plan alone time with my husband based on when my period comes… i.e. hotels without kids*” (S537). In extreme cases, people's menstrual cycles interfere with their professional life. S449 says “*I suffer from debilitating cramps that cause me to stay home from work, so I track my period to plan in advance as best I can*.”

Participants often started tracking when trying to get pregnant. S228 notes “*when I was younger I used to just remember. When I started trying for a baby, it became important to know my fertile times*.” People also change their tracking practices when they begin trying to become pregnant. For example, S18 previously tracked on her paper calendar, but later “*switched to apps when trying to get pregnant to get more symptom tracking*.” However, these processes can be “*too complicated to keep up with [post-partum]*” (S43).

Healthcare providers often ask a patient when her last period was as part of exploring her needs for more information and general attitude toward her body [7]. 25 women mentioned tracking to inform conversations with their healthcare providers. 6 other survey respondents began tracking at the recommendation of their doctor, including A668: “*it's great because it lets me record unusual symptoms and then I can remember them for my doctor visits*.” For many, the motivation is to know details related to their period when their doctor asks: “*my doctor asks, and I don't want to seem totally clueless about when it last was*” (S420). Women's menstrual cycles tend to become irregular pre-menopause [42] and immediately post-partum [32]. S181 is approaching menopause, and tracks “*so I can accurately tell my doctor just how irregular my cycles are getting*.”

For many women, tracking their menstrual cycle is an obvious and essential activity. I03 said, “*my mother just taught me [tracking] was the thing you did. I don't think there was ever any real explanation for why*.” S580 noted “*I didn't really think much about it. [tracking] was almost like a routine*.” Participants indicated the downside of not keeping track was too high to not know where they were in their cycle: “*it really sucks to be caught unaware*” (S48), “*I want to be prepared for it (emotionally and physically)*” (S393). This differs from other motivations typically considered in personal informatics. Perhaps closest is the idea that people tracking their finances seek out a *financial touch*, or a quick glimpse at data for awareness of their financial situation [21].

## How Women Track Their Cycles

We surfaced six methods and tools women use to track their cycle. Women (1) use phone apps, (2) use digital calendars, (3) write in paper diaries, (4) follow cues in their birth control, (5) notice symptoms, or (6) simply remember. We also report on women who do not track. Similar to tracking in other domains [12], women sometimes use multiple methods simultaneously and switch between methods. Table 2 shows the relative prevalence of each method in our survey participants. We again note that our recruitment approach led to a sample that over-represents women who track their menstrual cycle, especially women using technology to do so.

### Phone Apps

313 (47%) of our survey participants used an app on their phone to track their menstrual cycle. For some, finding an app to help track was the first method they thought of: “*common sense, there had to be an app for it. There's an app for everything*.” (S622). Others arrived at it after struggling to remember: “*my memory is awful, so I like keeping track using a dedicated app*” (S517) or because they wanted features other methods cannot provide: “*I used to use paper and pencil, but I like how the app can automatically predict the start of my next period*” (S41). For some, the switch to an app was a logical step after they got a smartphone: “*I used to use a calendar when I was a teenager…, [I] started using the app soon after I got a smartphone*” (S88).

In addition to serving as a diary of past periods, apps often use a woman's average cycle length to predict when her next period will occur and when she is ovulating. This information is typically conveyed in the home screen of the app, or sent in a push notification or email. Women often judge apps on the accuracy of these predictions. 302 app reviews mentioned how accurately the app predicted their cycle, with 65 reviewers describing apps as unhelpful due to inaccuracy.

Most apps support tracking factors beyond the timing of a woman's menstrual cycle, including information about the period itself (e.g., color, volume), factors predictive of period onset (e.g., cramping, mood), and other health-related factors (e.g., exercise, sleep). Some people appreciate tracking these factors. A94 was interested in understanding how her body reacts at different cycle phases: “*I have learned so much about my body/cycle from using the app, like how certain symptoms tell me I'm about to start my period… I always get moody around this particular time every month, etc*.” However, too many features can make apps more difficult to use: “*they keep adding more functionality, which is good but it makes things more cluttered*” (S91).

Women who are trying to conceive often find phone apps useful [28]. A395 says “*I got pregnant right away, and I believe it's because of Glow's algorithms and guided suggestions for optimizing chance of pregnancy*.” Apps often surface details which are helpful for becoming pregnant. S317 noted “*it gave me clearer information about ovulation*.” A1764 used Glow, which “*even tells you when to take a pregnancy test… It also reminds you to have sex on those important days*.” Alternatively, 19 women in our study reported using a phone app to avoid becoming pregnant. S381 said, “*I also have an active sex life and try to make a note of potential fertile periods as well as make sure I don't miss a menstrual cycle to try to help avoid pregnancy*.” In her app review, A233 notes “*you can breathe easy having unprotected sex after your fertile window (99% of the time)*.”

### Digital Calendars

83 (12%) survey respondents recorded their menstrual cycle via a calendar on their phone and/or computer. Most selected this method because it integrates with a tool they already use to manage life events and check frequently. S324 says “*I use Google Calendar for other appointments, so this made sense to keep everything tracked together*.” Some people tried using dedicated apps for menstrual tracking, but found “*it is too much trouble to use a separate app*” (S255, 5 others agreed). For many women, digital menstrual tracking followed when they switched scheduling from paper to a digital system.

In order to increase awareness of their typical cycle, some women record more information than when their period has occurred or will occur even without the support of an app. S274 records “two dots (..) for a heavy flow day, one dot (.) for a light flow day, and a star (*) for my estimated ovulation day.” 13 women mentioned marking a prediction of when their next period would occur in their calendar, based on when they got their period. S537 says, “*I count 28 days and put a dot so I know when to expect it next*.” S45 encodes her predictions: “*I enter an M into my iCal calendar for day 1… then an M? for when I think I'll get my period*.” 8 others, including S468, set up “*a repeating calendar event that tells me about when to expect it every month*.”

### Paper Diaries and Calendars

52 (8%) survey respondents use a paper diary or calendar to keep track, in much the same way that others use digital calendars. Many women use a paper system “*because I like using pen and ink*” (S142). Others, including S170, learned to track on paper, and do not want to go through the effort of switching to a digital system: “*In the 90's it was the only option. [I] got used to it. I'm too lazy to search for an app*.” Although we expected younger women would be more likely to use digital tools to track their menstrual cycles, Table 2 shows that younger respondents were no less likely to use a paper system. Several young participants reported they had adopted the paper-based tracking method their mothers used. S596 tracks in a notebook, and noted “*my mom uses this method and she recommended it to me*.” Conversely, some participants also mentioned exploring tools that their daughters had recommended. S537 currently tracks on paper, noting “*I just found out there is an app, my 16-year-old daughter uses it*.” S253 uses the P. Tracker app because “*my daughter suggested the app, and I find it easier than writing it on the calendar*.” Tracking methods are shared both from mother to daughter and from daughter to mother.

### Following Hormonal Birth Control

Pill-based hormonal birth control is typically in packs of 21 or 28 pills [40]. Both cases contain 21 days of pills with active hormones, with a woman's period typically arriving after finishing these pills. The 28-day packs contain one week of placebo pills, which are typically visually distinct, to sustain the daily habit of taking a pill. The NuvaRing [30] follows a similar cycle. A woman inserts it into her vagina for three weeks, during which it releases hormones preventing ovulation. She then removes it for one week, at which point she experiences her menstrual period. Other hormonal birth control pills have a longer cycle, with months between placebo pills and thus the arrival of the period (e.g., Seasonale [31]). These longer cycles are otherwise similar.

The women in our study who were using these types of pill-based birth control often kept track by simply noticing how far they were in their packs. S446 says, “*when my pills are gone for the month, I know my period is coming*.” For S21, “*the approach of the [placebo] brown pills signal the approach of my period*.” S520 notes the ease of this method, saying “*it requires no additional effort since I would already be taking my birth control*.” Participants with a NuvaRing typically used another method to track when they needed to insert or remove the ring. For example, I8 said “*when I was on the NuvaRing… I used a website called Bedside that would send me a text message about ring in and out*.”

### Noticing Early Symptoms and Physical Changes

Many women notice physical or emotional changes which typically align with the arrival of their period, from bloating or breast soreness to irritability or fatigue [36]. Some survey respondents paid close attention to these changes, using them to predict the arrival of their period. For S146, the physical changes tell her to be prepared: “*from the onset of soreness I give myself a few days, and then I'm on high alert*.” S639 mentioned she has an irregular period, and noticed “*I get depressed and moody a few days before I start… I am able to recognize these emotions as a period coming*.”

### Remembering

Women also keep track simply by remembering when their last period occurred: “*I just remember the date when my last period started and count ahead ∼25-30 days*” (S468). This method is most effective for women who have especially regular periods and can easily predict when their next one will be. Others instead try to remember what they were doing when their period occurred, such as S472: “*I try to remember what I was doing the day it started the prior month and extrapolate from there*.” 11 survey respondents relied only on their memory because “*it's the least conspicuous method*” (S221). 19 others simply could not remember to use another system: “*I'm not good at remembering to note the dates of my period in apps or paper calendars”* (S132).

### Not Keeping Track

89 survey respondents indicated they did not track at all. 17 of those 89 later described tracking in line with the styles above, so we reclassified them. 25 respondents do not currently have a period for various reasons, including birth control: “*Mirena IUD made it stop*” (S118, 14 others), menopause (6 respondents), surgery: “*procedure to reduce excessive bleeding during periods was very successful… I stopped having them*” (S293, 1 other), a current pregnancy (1 respondent), or “*intense sports*” (S589).

The remaining 47 participants did not keep track, but many still prepared for their period. 26 participants mentioned having supplies always available: “ *[I] keep tampons at home and in my car all the time*” (S540) or beginning to carry or use supplies preemptively: “*if I think it may be coming soon, I will buy a box of tampons and keep some in my backpack*” (S175). 11 participants reported they do nothing to monitor or prepare for their period. This lack of monitoring is sometimes problematic, such as for S107: “*I do nothing at all. It leads to quite a few ruined pairs of underwear*.”

## Implications For Design in Menstrual Tracking

Women described many challenges with how they track their menstrual cycle. These challenges illustrate problems in the design of technology to support menstrual tracking.

### The Importance of Accuracy for Prediction

**Figure 1:**
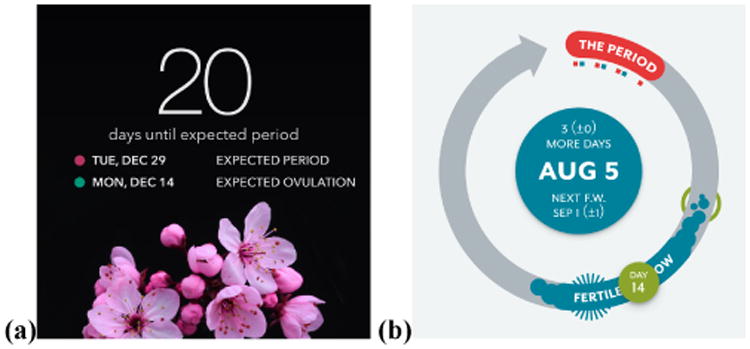
Phone apps predict a woman's next period or ovulation. Life (a) surfaces this prediction as a point estimate, while Clue (b) provides a range of potential dates.

Predictions of where women are in their cycle must be accurate to be useful. In the case of predicting the first day of menstruation, women want to know for planning purposes. For example, S399 relies on her app notification to not forget and to be prepared: “*it conveniently gives me notifications two days early, otherwise I would always forget*.” Menstrual tracking apps vary in how they present these predictions. Some apps present the prediction as a single-day estimate of when a woman's period or ovulation will begin (Figure 1a), others present the prediction as a range of possible affected days or include errors in their estimates (Figure 1b).

**Figure 2:**
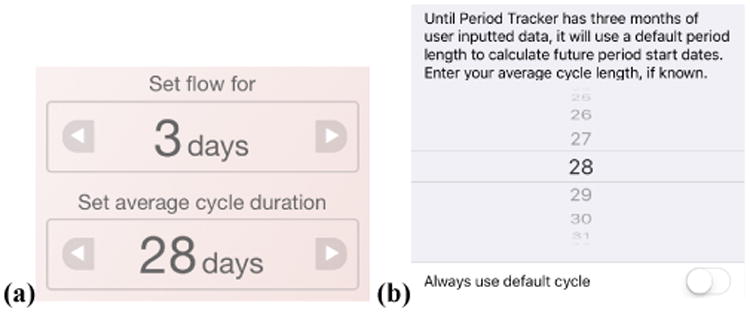
Apps, such as My Cycles (a) and Period Tracker (b), typically ask for average cycle duration and flow length to aid in prediction. Although this prediction may be later aided by journaled data, it is not resilient to variations due to irregular cycles, stress, birth control, and even forgetting to journal.

Similar to results in other domains [12], women abandon inaccurate menstrual tracking apps and search for more accurate alternatives. However, predicting the first day of a period and time of ovulation is challenging. 90% of women have a period every 24 to 38 days, which clinicians define as a “normal” cycle frequency [15,42]. This range is broad and ignores the 10% of women whose cycles are more or less frequent. Cycle regularity varies as well. Some women have a fixed-length cycle, while others can vary by days or even weeks [15]. To aid in this prediction, one of the first questions apps often ask women is to enter their average cycle length and flow duration (Figure 2a). This information is typically used to predict the first day of a period, which is later informed by the data recorded in the app (Figure 2b).

Many women find current modeling assumptions about stability and regularity insufficient for predicting their menstrual cycle. A1638 has an irregular cycle and found her app assumed some regularity: “*The whole reason I need a period app is due to my extremely irregular periods… For someone whose days vary it's hard to use. Sometimes my periods are longer than normal and the app assumes I must have forgotten to hit the ‘period end’ button and does it for me*.” S369 notes that a particular cycle may be abnormal as well, which impacts later prediction: “*If something out of the ordinary happens (Plan B, particularly stressful month), it doesn't take that into account when predicting your next period*.” For S188, this effect snowballs: “*if I'm stressed I usually run a week late. That messes up the accuracy of my next cycle prediction… which then makes me more stressed*.”

Models additionally fail to keep up with life changes. For example, A81 was “*very faithful at keeping track of her periods up until I got pregnant*”, but the app no longer works for her: “*a pregnancy, baby, and year and a half of breastfeeding later, the app thinks my normal cycle length is about every 700 days!*” She does not want to switch apps and lose her data: “*Please add a pregnancy mode :) I don't want to have to start over!*” However, apps also poorly account for more routine changes, such as a woman switching her method of birth control. S398 started using an IUD, noting “*the length of my cycles as reflected on the app is completely out of sync. It would be nice to have options that account for the fact that I still have a standard menstrual cycle, despite not having a typical period every month*.”

Similar to barriers in other domains [12], people often forget to log their period. Most apps require logging both when a period starts and when it ends. Logging the end can be particularly difficult to remember: “*it's easy to forget about towards the end of your period, so the actual cycle is often not completely accurate… but that's more of a user failing ;)*” (S59). Beyond the annoyance of having an incomplete or inaccurate record, S65 noticed it limited how well her app predicted her period later: “*I sometimes forget to enter my data one month, which skews the data for the next month*.”

#### Recommendation

Given how crucial prediction accuracy is to menstrual cycle tracking, designers should evaluate additional techniques for modeling and communicating predictions about a woman's cycle. Reasons for tracking likely correspond to different prediction needs. A woman tracking to avoid becoming pregnant would probably prefer her app overestimate her ovulation window. A woman trying to become pregnant may instead prefer a conservative estimate of her ovulation. For both ovulation and period arrival, designers should consider and evaluate interfaces that present probabilities as an alternative to unreliable binary predictions.

Current app predictions do not sufficiently account for life experiences that can impact a woman's menstrual cycle (e.g., stress, exercise or diet changes, some emergency contraceptions). At minimum, apps should allow women to record when the app's prediction falls out of line with when their period actually occurs, and should use this information to improve future prediction accuracy. Designers should consider how to account for the variability caused by irregular cycles and life changes. For example, designs could occasionally ask women to offer their own prediction, using that opportunity to identify changes.

### Gendered Design and Nonconformity

**Figure 3:**
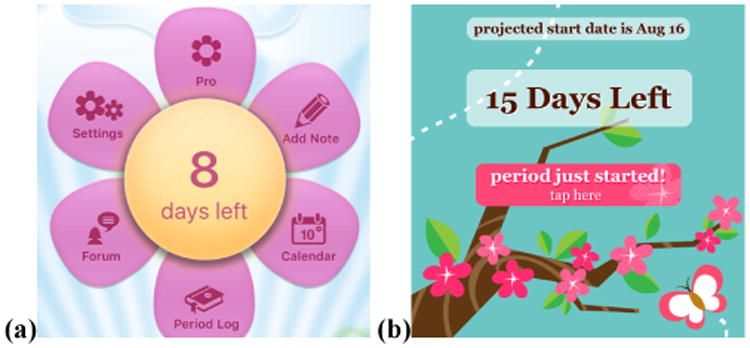
Period tracking apps often employ feminine, flowery, pink aesthetics. (a) is Period Diary, (b) is P. Tracker Lite.

Similar to a trend noticed by Peyton et al. in the context of pregnancy apps [34], the menstrual tracking apps we reviewed tended to use stereotypically feminine attributes, such as the interface being predominantly pink or using flower and heart images (Figure 3). S98 found the design of her app insulting: “*they have tried to make it ‘feminine’ by adding flowers… It makes me feel like you are trying to ‘dumb it down’ for me. Why can't keeping track of my menstruation be a professional and organized task?*” (14 other survey respondents agreed). I09 tried a couple of different apps, finding: “*a lot of them just felt kind of condescending or like they were designed by dudes who were designing what they thought a woman would like*.”

Our sampled app reviews often mentioned the femininity of apps. 13 reviews appreciated the femininity, including A194 “*I love the way it includes necessary info in a fashionable girly way. :-)*.” However, 44 app reviews considered femininity a more negative design trait, and valued when they found more gender-neutral designs: “*I spent quite a while trying to find [a period tracking app] that wasn't pink and/or flowery, but I finally did and I'm impressed*” (A229 reviewing the app Clue, Figure 1b, 4b). 38 of these reviews were for the Clue app, which was described as a “*gender neutral*” alternative (A740).

**Figure 4:**
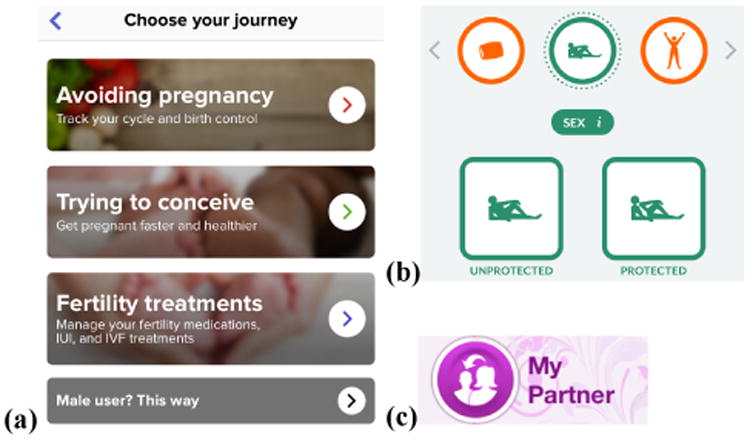
In Glow (a), people who identify as male are directed to an alternate view of the app. Clue's iconography (b) suggests a male sexual partner, while the iconography in My Period Tracker (c) implies a female sharing data with a male partner.

Beyond pink and flowery interfaces, app designers often assume that the person tracking their menstrual cycle identifies as female (Figure 4a). A841 struggled to “*find a period tracker that didn't misgender me*”, before settling on Clue. S365 identifies as male and also uses Clue, noting *“it's hard to find tools that work for me! [Clue] uses gender neutral language… it's also not focused on pregnancy, which I'm not interested in at all*.” Tracking ovulation is often a major feature of menstrual tracking apps, appearing on the main app page alongside period information (Figure 1). A1086 has no need or interest in seeing this information: “*I wish it were less catered to birth and family planning… I suppose I'm in the minority since I'm asexual*.”

Furthermore, menstrual tracking apps often enable logging sexual activity. This feature is often designed to help women track sex alongside ovulation, either to become or avoid becoming pregnant. However, women track sex for reasons unrelated to fertility. I06 appreciated logging to check how her relationship is going. Her app puts a heart on the calendar whenever she logs sex, which she appreciates: “*I can tell if we're off or something, so I can look and can get back on track… I just like looking back and seeing the little hearts everywhere… it's fun. I like to see how often we have sex*.” A1928 notes “*to me, this is just as important!* … *it's hard sometimes to realize/remember how long it's really been*.”

Menstrual tracking apps often assume a sexual or relationship partner is male. For example, Clue provides two options for logging sex, with both icons suggesting a male partner (Figure 4b). S240 was particularly bothered by this, noting “*sex options assume sex with a man, and reminder of ovulation cycle both remind me I am not a ‘normal’ woman whenever I use the app… but it's not overly pink so I deal*.” A232 agreed, noting “*as someone in a same sex relationship protection isn't really a concern, but I would like to keep track of my ‘activity’ levels*.” Other apps enable sharing of period and fertility data with a partner. This partner is sometimes implied to be male, such as in the iconography for My Period Tracker (Figure 4c). The partner cannot log their own menstrual cycle in some apps, despite the desire for the couple to track together. A397 notes in her review of Glow, “*it's an amazing app, but it's meant for straight couples*.”

#### Recommendation

More people can benefit from tools with gender-neutral themes that do not assume gender in labels, iconography, and functionality. Similarly, when logging sex, apps should be careful not to assume the sexual orientation or gender of either the person logging or their partner(s). Logging sex and reporting on fertility can be desirable for some women and uncomfortable for others. Designs should consider including features to support tracking sex and fertility, but should also support disabling those features.

### The Discreet Nature of Menstrual Cycle Tracking

Women often treat menstruation as a personal matter they do not want to disclose to others [24]. Women who track their menstrual cycles with technology reported concern about accidentally disclosing where they are in their cycle when showing their calendar to friends or coworkers. In particular, people often share their calendars permanently or temporarily, which can cause discomfort for women who have information about their menstrual cycle on their calendar. S188 says, “*It's weird having it on my calendar so publicly, I wish it was there but somehow more secretly*” (7 others expressed a similar sentiment). To combat this disclosure, 21 survey respondents described using a method to “*encode the events*” (S13) to keep the information private. Women most commonly encode with a simple symbol, “X” or “.”, instead of describing the event. Others aim to be more discreet, such as S28: “*I use ‘rrrrr’ for period… Secret, only I know what ‘rrrrr’ means*.” S409 describes her method: “*Luckily, I know an esoteric language, so I write [when my period is coming] in that different language*.”

Some women prefer using a dedicated app because of privacy, including S192: “*keeping info in an app instead of written on my calendar gives me greater privacy*.” However, women were still concerned about revealing personal information when these apps are used in front of others. Privacy concerns are perhaps exacerbated by app designs. A781 “*used to be embarrassed when other people looked at my phone and saw a bright pink tracking app*”, which led her to switch to Clue: “*this design lets me feel more secure in letting other people handle my phone*” (17 others agreed). S102 “*renamed the app ‘tracker’ on my phone instead of ‘period tracker’*” to make the app more discreet. A76 agreed, suggesting “*a less obvious title would be awesome*” for the app “Period Tracker.” 9 app reviewers of 5 different apps appreciated their app's discreetness. A1503 said, “*no one will guess what it is because it looks like an average app*.”

Although notifications can help women remember when their period is coming, they also conflict with the desire for discreetness. S440 has trouble remembering to monitor her period: “*you do still have to remember to use it*”. She suggests reminders could help, “*but that makes it a little less discreet. As is, the app could still be more discreet*.” S283 disabled the notifications “*since they're kind of personal, but as a result I sometimes forget to enter the period in and have to try to remember when it was later*.”

#### Recommendation

Apps for menstrual cycle tracking should design for customization. Designs should either be discreet by default or provide a neutral non-obvious interface option. Such discreetness is similar to Consolvo et al.'s design choice in UbiFit Garden, abstractly encoding physical activity data as a garden [9]. Many women desire app color schemes, icons, names, and notifications that do not draw attention from others who casually glance at their phone. Designs should support disabling and customizing notifications. Any decision to show tracked data to someone else should be explicitly triggered.

### Supporting Varied and Changing Reasons for Tracking

Apps support many of the motivations women have for tracking their menstrual cycle (Figure 4a). Supporting many tracking motivations provides benefits, including supporting people when their goals change. S148 “*initially started because I was fairly irregular and wanted to find any trend I could*”, but now has different goals: “*now I track to make sure I'm not missing my period – a pregnancy check, basically*.” S318 originally tracked for awareness: “*before, it was to know when it would next come*”, and is now trying to become pregnant: “*more recently, it has been to know when I'm ovulating for conception*.” In practice, apps for menstrual cycle tracking are not wholly successful at supporting varying goals. S127 has tracked for both health and fertility, noting “*some [apps] have features for health and some have features for fertility planning… to make the most, I have used various apps at the same time and entered data into them twice*.”

One option to better support these goals is to include more information and options relating to fertility, pregnancy, and post-partum. However, some women believe apps already focus too much on these areas. S467 felt her app is “*clearly trying to support my getting pregnant (which is not my intent) and not just agnostically for tracking*” (8 others agreed). A1936, who just started having her period, felt the ovulation information in Clue was not relevant to her: “*I would like it if they made a kid's version because idc [I don't care] about fertile!! I'm too young!!*”

Moreover, ovulation information can be uncomfortable for someone who has struggled with infertility. S104 writes: “*my app shows predicted ovulation. I wish it didn't. We dealt with infertility and extensive treatments for 6 years. I am no longer trying to get pregnant and I don't like the reminder of TTC [trying to conceive] or the tiny glimmer of hope that maybe by magic this will be the month when a miracle happens*.” She now primarily tracks “*so I know when to expect [my period]*”, but the app she uses presents information about ovulation despite her discomfort. Two others described the same concern, saying tracking is a “*constant reminder of trying to conceive and not succeeding*” (S131).

#### Recommendation

To resolve the problem of varied and changing motivations, menstrual cycle tracking apps can support reconfiguring the main interface. For example, apps could provide a different view when someone begins trying to conceive, utilizing their menstrual cycle history to accurately predict when they will ovulate. Alternatively, apps could enable exporting menstrual cycle history or could share data among a collection of apps, so another app designed for a woman's new goal could utilize previously logged data. Further, being able to retrieve tracked data outside the app to share with a healthcare provider or when transitioning to a new phone is often helpful. Unfortunately, personal informatics data are largely siloed within a specific tool or company [26]. Some menstrual tracking apps enable data backups for switching or resetting devices, but not consistently: “*I went from an iPhone system to an Android and nothing was saved so I manually inputted what I had*” (A782).

### Menstrual Tracking Alongside Other Tracking

Some menstrual tracking apps support journaling other aspects of wellbeing, such as mood, headaches, insomnia, and sexual activity. Participants noted these aspects sometimes aligned with their menstrual cycle. I04 noted, “*I was consistently being depressed right before it happened. I would be really unhappy and grumpy, and then I'd get my period*.” Tracking her period alongside her mood helped better explain her mood: “*keeping track of it, then, when I remembered to look at a calendar, I'd be, ‘oh, this is why I feel like this’*.” In the notes field of her menstrual tracking app, I05 tracks “*whether I started having some kind of symptom like headaches or bloating… just to see if anything correlated*.”

Some participants were particularly interested in aligning their menstrual cycle data to other tracking. I09 said “*I have noticed a connection between my resting heart rate and my menstrual cycle*.” However, identifying the trend was hard: “*neither my menstrual cycle app nor the Fitbit allow me to export the data, so I have to manually type the data into a spreadsheet to see the trend. I'd love it if I could export the data, or if I could track both via one app*.” Some apps readily support data integration and export. A596 says “*I really like how my FitBit info can be synced with Glow*”, and A219 appreciates that her app “*integrates with Apple Health*.”

#### Recommendation

Menstrual cycles affect and are affected by other aspects of women's lives. Apps for tracking menstrual cycles should support women in identifying these connections. Apps could include the ability to journal concerns and correlations alongside menstrual cycles, such as mood and stress. Apps can further support developing open-ended categories when a woman wants to journal something that is unsupported. Apps should allow tracked data to be exported, ideally supporting interoperability with other popular platforms for tracking health data (e.g., Fitbit, Apple Health, MyFitnessPal). We note some apps include these capabilities already, but their implementation should be widespread.

### Discussing Tracked Data with Doctors

For many health concerns, patients report dissatisfaction with how providers engage with the data that patients bring to appointments [8]. Our participants similarly noticed that doctors tended not ask about their data, particularly when their health was normal. I07 said “*none of [my doctors] asked, ‘oh, can I see your written calendar?’… just sort of my general take of it… that was as much as they really seemed to care*.”

On the other hand, when addressing a specific health concern, healthcare providers appreciated having data about the patient's menstrual cycles. I11 used a spreadsheet to track everything she and her husband tried while undergoing fertility treatments. Whenever she visited a new doctor, she would summarize the spreadsheet and give them a print-out of what they had tried: “ *[the doctors] don't have much time, I felt like if I had just printed off the massive spreadsheet and handed it to them, there's pretty much zero chance they would have looked at it… I really tried to just summarize it, and give them a 1 or 2-page sheet… they loved that… otherwise they have to go through my record, which is a massive packet of stuff*.” I06 has an especially irregular period and occasionally bleeds outside of her primary cycle. Her doctor finds this information useful: “*when we're trying to figure out whatever was going wrong, it was very helpful for her to see the start and end dates and how long it had been in between periods, and how long the period had lasted… I would consolidate what I have in a Google document… I always synthesize it*.”

#### Recommendation

Designs should offer ways to summarize and export data for a healthcare provider. Prior work notes summaries of patient-generated data should be concise, follow a standard format, and integrate into digital health records [8].

## Discussion

Our findings echo problems people encounter in many personal informatics domains. People migrate between goals and tools for tracking their menstrual cycles as their needs change. Personal tracking tools fail to support such transitions and do not enable data migration between tools [12]. Gendered or heteronormative designs of such tools exclude people from using them and alienate others who continue to use them.

Li et al. define personal informatics tools as “*those that help people collect personally relevant information for the purpose of self-reflection and gaining self-knowledge*” [25]. Although this definition is broad, most research has focused on tracking behaviors and outcomes in support of behavior change or tuning. Menstrual cycle tracking reminds the community that tracking one's experiences, rather one's behavior, can lead to important self-knowledge.

Personal informatics research has also focused on domains where people have some amount of *control* over the behavior being tracked, such as spending, physical activity, or food choices. In menstrual cycle tracking, however, women primarily *observe* what they are tracking, with little or no control over how and when it will occur beyond hormonal regulation through birth control. Rather than trying to change the outcome being tracked, women track to learn how to adjust their thoughts and behaviors around it. In other domains where the person tracking lacks control, such as tracking pain and allergic reactions, observing the event can help explain symptoms and even identify causes.

People tracking physical activity or weight data often focus on overall trends, rather than the accuracy of any individual data point [19,44]. If overall trends are sufficiently accurate to plan and adjust behavior, accuracy in individual data points is less of a concern. For menstrual cycle tracking, however, considering the overall trend is insufficient. Women plan around the specific predictions provided by their app, which rely on the accuracy of past data points. People's needs surrounding accuracy in personal informatics vary by domain and how the data is being used. Designers of personal informatics tools should work to build an understanding of acceptable accuracy [20] for the domain in which they work and the specific type of insights their tools will offer.

This paper aims to promote a conversation surrounding menstrual cycle tracking within HCI. The tracking practice is extremely widespread, spanning much of the lifetime of much of the population. When we posted our survey to social networks, we were overwhelmed by the amount of interest the study received. On Facebook, participants expressed appreciation that this topic was receiving attention, including “*hats off to [researchers] for tackling this*” and “*can't wait until they post their results*.” Others lamented the current state of technology: “*I've tried 4 apps. They all suck… I would think a creative woman would've created something better by now…*” Menstrual tracking tools are important, but for many people current applications fall short. Designers, engineers, and researchers can do more to make these tools more inclusive and to reflect the changing needs of women.

## Conclusion

We contribute an understanding of why and how women track their menstrual cycles with an eye towards the problems they encounter with current tracking methods. Designs should avoid gendered coloring, iconography, and text to both be discreet and inclusive of people's gender identities and sexual orientations. Designs should support the varied reasons people track their menstrual cycles, and should support migration between tracking goals or tools. Women rely on accurate predictions from their menstrual trackers. Our research emphasizes the importance of designing for inclusion, acceptable accuracy, and discreetness in personal informatics.

## Supplementary Material

1 - Demographics

2 - Survey Protocol

3 - Interview protocol

4 - Info Sheet - Adults

5 - Info sheet - Minors

6 - Recruitment messages

7 - Methods details and codebooks

## Figures and Tables

**Table 1:** We collected data from three sources: app store reviews, a survey of women's practices, and follow-up interviews.

	App Reviews (2000 reviews)
iPhone apps	Clue (200), Eve (150), Glow (200), Life (200), P. Tracker (200) Period Diary (170)
Android apps	Clue (160), Glow (160), My Days (120), P. Tracker (160), Period Calendar (120), Pink Pad (160)
	**Survey Demographics (687 people)**
Gender	656 female, 11 nonbinary, 2 male (18 no answer)
Orientation	478 heterosexual, 91 bisexual, 21 homosexual, 8 asexual, 11 fluid, 38 other queer (40 no answer)
Age	min 13, max 60, mean 28.0, median 27 (14 no answer) 103 <18, 66 18-23, 265 24-29, 172 30-39, 67 ≥40
Apps used (313 people)	Clue (104), P. Tracker (89), monthlyinfo.com (10), Life (10), 33 apps with less than 10 people each
	**Interview Demographics (12 people)**
Gender	10 female, 1 nonbinary (1 no answer)
Orientation	4 heterosexual, 3 bisexual, 2 homosexual, 1 fluid, 1 other queer (1 no answer)
Age	min 17, max 40, mean 28.6, median 28 (1 no answer) 1 <18, 1 18-23, 4 24-29, 4 30-39, 1 ≥40

**Table 2:** The majority of survey respondents used phone apps to keep track of their menstrual cycles.

Age	N	Phone app	Digital calendar	Paper calendar	Birth control	Early symptoms	Remembering	Do not track
<18	103	50%	6%	9%	5%	9%	27%	6%
18-23	66	48%	9%	8%	17%	8%	15%	8%
24-29	265	43%	12%	6%	18%	6%	20%	9%
30-39	172	51%	17%	9%	6%	6%	14%	8%
>40	67	34%	12%	9%	6%	9%	12%	20%

Overall	687	47%	12%	8%	12%	7%	19%	11%

## Footnotes

1 We use the gendered term “women” in this paper to refer to anyone who has, or has previously had, a menstrual cycle. This follows the general use of the term “women's health” in the HCI community to discuss health issues around pregnancy, menstruation, menopause, and breast cancer. We acknowledge not all people who have a menstrual cycle identify as women, and not all people who identify as women have a menstrual cycle.
