# Supplementary figures and images for: Examining Menstrual Tracking to Inform the Design of Personal Informatics Tools
Source: Proc SIGCHI Conf Hum Factor Comput Syst. Author manuscript; Available in PMC 2017 May 15. (PMC5432133; doi:10.1145/3025453.3025635)

# Participant demographics

## Age

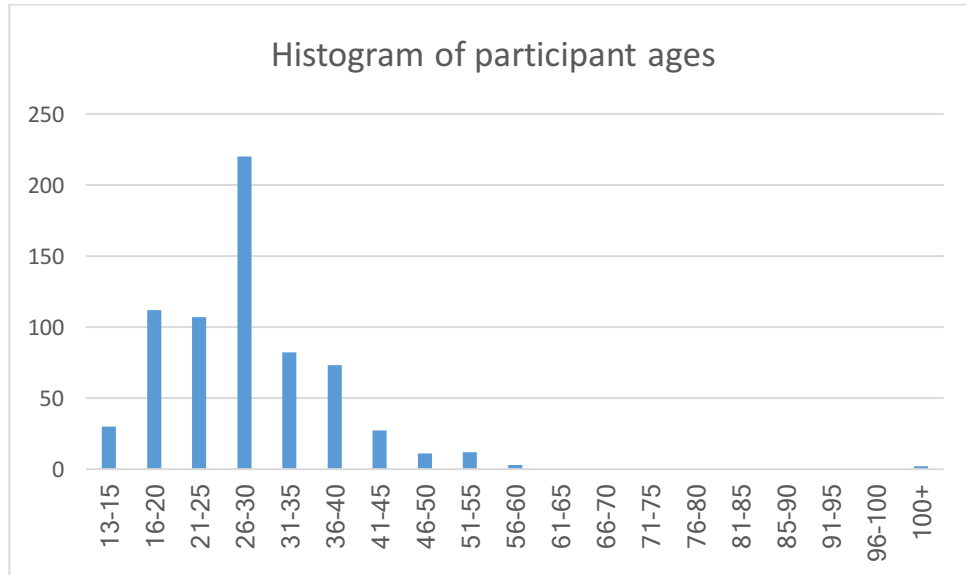

## Race

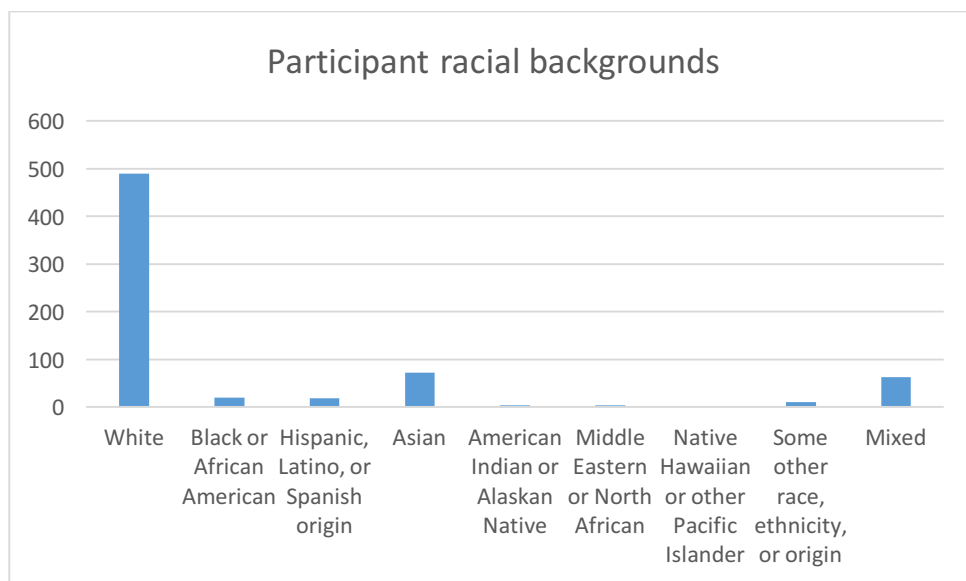

## Income

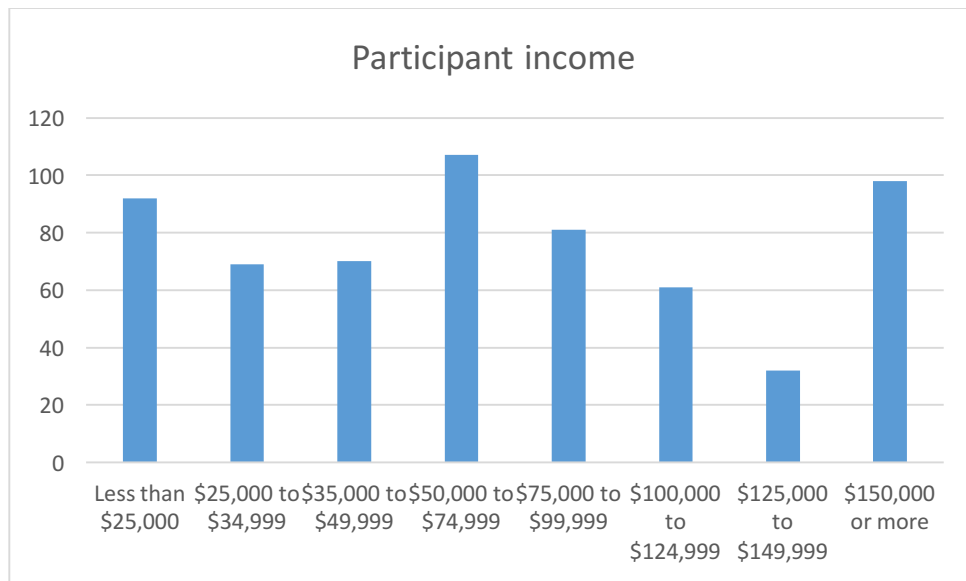

## Device ownership & access

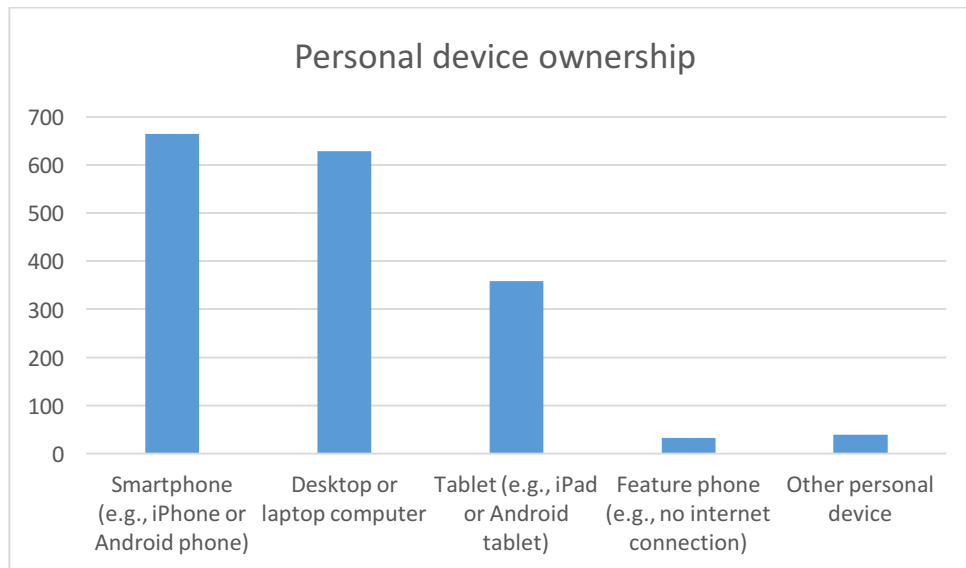

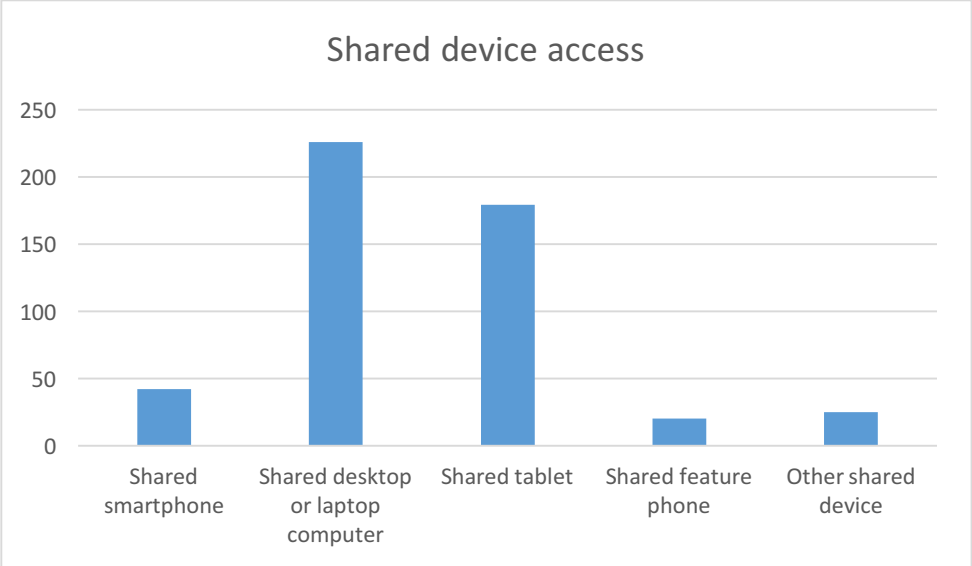

Supplement: 1 - Demographics [file NIHMS855306-supplement-1_-_Demographics.pdf]
