# Supplementary material for: Examining Menstrual Tracking to Inform the Design of Personal Informatics Tools
Source: Proc SIGCHI Conf Hum Factor Comput Syst. Author manuscript; Available in PMC 2017 May 15. (PMC5432133; doi:10.1145/3025453.3025635)
Supplement: 2 - Survey Protocol [file NIHMS855306-supplement-2_-_Survey_Protocol.pdf]

# Online survey

Note: this is a simplified version of the survey, with branching logic streamlined. The version presented here is what most participants experienced, with slightly different logic for people who did not have a menstrual cycle or were not tracking or monitoring it.

## Screening

- Have you had your first period? [yes/no]
- How old are you? [less than 13 years old/13-17 years old/at least 18 years old]

## Consent

(study description and information sheet provided)

- I have read the description of the study above, and consent to participating. [yes/no]

## Introduction

- As best as you can remember, how old were you when you first got your period? [number]
- Do you try to keep a record of when you period has happened? [yes/no/I do not get a period anymore]
- Do you try to predict when your next period will be? [yes/no/I do not get a period anymore]
    - How do you know when your next period will be? [app/calendar/paper system/remember]
    - (if app) What app do you use to keep track? [text field]

## Tracking behaviors

- Why do you keep track of your period? [text field]
- Please describe your method for keeping track of your period. Describe your method as if a 13 year old asked you how you keep track. [text field]
- How did you come to use this method? What led you to use this method? Have you tried other methods? If so, what have you tried? [text field]
- For how long have you used this method? [text field]
- What do you like about this method? Aim for listing two or three things you like. [text field]
- What do you dislike about this method? Aim for listing two or three things you dislike. [text field]

## Period frequency and regularity

- Are you on a birth control that impacts the regularity and/or duration of your cycle? [yes/no]
    - How does your birth control impact your period? [text field]
- On average, how long is your period cycle? [<24 days, 24-38 days, >38 days, I don't know]
- On average, by how many days does the arrival of your period vary? [my period doesn't vary/2-5 days/6-20 days/>20 days/I don't know]
- On average, how long does your period last? [<4.5 days/4.5-8 days/>8 days/I don't know]

- Is there anything else notable about the frequency or regularity of your period? [text field]

## Demographics

- How old are you? [number]
- What is your race? Select all boxes that apply below. Please note you can select more than one. [White/Black or African American/Hispanic, Latino, or Spanish origin/Asian/American Indian or Alaska Native/Middle Eastern or North African/Native Hawaiian or Pacific Islander/Other]
- What is your annual household income? (if living with roommates, personal income) [<$25k, $25-34.999k, $35-$49.999k, $50-74.999k, $75-99.999k, $100-124.999k, $125-149.999k, >$150k]
- What digital devices do you personally own? [smartphone/desktop or laptop computer/tablet/feature phone/other]
- What digital devices do you share with other people? [smartphone/desktop or laptop computer/tablet/feature phone/other]
- What is your gender identification? [text box]
- What is your sexual orientation? [text box]
- [raffle opt-in and email field]
- Are you willing to be contacted for a one-hour interview over the phone or Skype? We can compensate interviewees with an additional $20 gift card to Amazon or Starbucks. [yes/no]
