# Supplementary material for: Examining Menstrual Tracking to Inform the Design of Personal Informatics Tools
Source: Proc SIGCHI Conf Hum Factor Comput Syst. Author manuscript; Available in PMC 2017 May 15. (PMC5432133; doi:10.1145/3025453.3025635)
Supplement: 3 - Interview protocol [file NIHMS855306-supplement-3_-_Interview_protocol.pdf]

Interview Protocol

Intro Script

Hi, I'm ______. _____ is also listening to take notes, but may occasionally ask follow-up questions (say hi).

We're conducting a study about how women monitor their menstrual cycles. We wanted to talk to you more about your habits. This interview will last no more than an hour. Can we record audio? You can choose not to answer any question, and can stop the interview at any time.

Intro and Icebreakers

- What made you decide to monitor your period? (be prepared to ask for more detail)
- How do you monitor your period?
- How did you come to use this method for monitoring your period? (probe for… recommended from others? Search on a store? Read about online?)
- Have you ever discussed your method with others? Who have you discussed it with? (friends or family, doctor, spouse/significant other; probe for privacy--do you have any privacy concerns about discussing your method with others?)
- What do you particularly like about your method? (probe for discreet and privacy--do you have any concerns about how visible your method is to others?)
- What do you dislike about it? (probe for discreet and privacy, particularly if there are challenges around heteronormativity)
- [if people bring up how burdensome a method is] what about your method do you find burdensome?

Period Monitoring Lifecycle

- Have you tried other methods for monitoring your period?
- Have you considered trying any other methods?
  - [if yes to trying others] What do you prefer about your current method for monitoring your period over the others you've tried? What did you dislike about the other methods?
  - [if yes to trying others] What else have you tried?
- How has your experience monitoring your period changed over time?
- Have your needs for monitoring your period changed at all? (probe for pregnant/not pregnant if that's a thing they mentioned in their survey)

Uses of Data

- What do you do with the data you get by monitoring your period? (probe on motivations, especially if they don't seem to do a lot of reflection or prediction; what motivates you to monitor your period?)
- [for people who share with your doctor] How do you show your data to your doctor?
  - Have you changed how you monitor your period because you're showing it to your doctor?
  - What is it you show your doctor? (possible examples include raw data of when it was or how it was, app screenshots, insights you gained…)

- Do you monitor anything else? Some examples of things people commonly monitor are physical activity or other health, foods consumed, finances, location…
    - [if yes to the above] How does your experience monitoring your period relate to these other forms? Do you find it more or less important? Do you ever try to find relationships across the different things you monitor?
    - [if no to the above] Would you consider monitoring anything else? If you could monitor something with little/no effort, what would you want to monitor?
