# Supplementary material for: Examining Menstrual Tracking to Inform the Design of Personal Informatics Tools
Source: Proc SIGCHI Conf Hum Factor Comput Syst. Author manuscript; Available in PMC 2017 May 15. (PMC5432133; doi:10.1145/3025453.3025635)
Supplement: 4 - Info Sheet - Adults [file NIHMS855306-supplement-4_-_Info_Sheet_-_Adults.pdf]

INFORMATION SHEET

Understanding how people use period apps

Researchers: Daniel Epstein, Principal Investigator: Graduate Student, Computer Science & Engineering.
Contact info: depstein@cs.washington.edu, 703-599-7653
James Fogarty: Associate Professor, Computer Science & Engineering
Julie Kientz: Associate Professor, Human Centered Design & Engineering
Sean Munson: Assistant Professor, Human Centered Design & Engineering

PURPOSE OF THE STUDY

We are studying how people use applications to track their menstrual cycle. People have used calendars and journals to track their cycle since long before smartphone applications came about to aid in the process. Understanding how women track their periods with and without technology can help inform the design of future self-tracking applications, for period tracking and beyond.

STUDY PROCEDURES

We are conducting a survey of people who are currently or have previously tracked their menstrual cycle. We expect this survey to take no more than 30 minutes to complete. You are not required to respond to any question that makes you feel uncomfortable, and may end your participation at any point.

RISKS

You may experience some discomfort with completing the survey, particularly given the topic area. There is a risk that information about your sex life or reasons for tracking periods related to sexual activity may be disclosed, whether accidental or otherwise. This information may be sensitive in nature and could pose a risk if disclosed. You may skip any question or end your participation at any time. We do not anticipate any physical risks to participants.

BENEFITS OF THE STUDY

You will not directly benefit from taking part in this study. However, this research will help inform the design of period tracking apps.

CONFIDENTIALITY OF RESEARCH INFORMATION

Taking part in this study is voluntary, and you can stop at any time. The data collected in this study will be held confidential.

All of the information you provide will be confidential. However, if we learn that you intend to harm yourself or others, we must report that to the authorities.

OTHER INFORMATION

You may refuse to participate and you are free to withdraw from this study at any time without penalty or loss of benefits to which you are otherwise entitled.

Survey respondents who provide an email address will be entered into a drawing for a $100 gift card to Amazon or Starbucks (your choice).

You may contact the research team phone (703-599-7653) or email (depstein@cs.washington.edu) if you have any questions about the study or if you feel you have been harmed by participating. If you would like to know more about your rights as a research

participant or if you have a concern, please contact the Human Subjects Division at UW at (206) 543-0098.
