# Supplementary material for: Examining Menstrual Tracking to Inform the Design of Personal Informatics Tools
Source: Proc SIGCHI Conf Hum Factor Comput Syst. Author manuscript; Available in PMC 2017 May 15. (PMC5432133; doi:10.1145/3025453.3025635)
Supplement: 5 - Info sheet - Minors [file NIHMS855306-supplement-5_-_Info_sheet_-_Minors.pdf]

UNIVERSITY OF WASHINGTON
Information Sheet for Minors

Study of how people use period apps

Researchers: Daniel Epstein, Graduate Student, Computer Science & Engineering (contact info: depstein@cs.washington.edu or 703-599-7653)
James Fogarty, Associate Professor, Computer Science & Engineering
Julie Kientz, Associate Professor, Human Centered Design & Engineering
Sean Munson, Assistant Professor, Human Centered Design & Engineering

<u>Researcher's statement</u>:

My name is Daniel Epstein.

We are asking you to be in a research study because we are trying to learn more about how you track your period. For example, do you use an app? Do you keep it on your calendar? Do you remember when it is?

If you agree to be in this study, we'll survey you about how you keep track of your period.

You might feel uncomfortable completing a survey about your period. Your responses might be sensitive, such as why you're tracking your period. This could be uncomfortable or embarrassing if disclosed, accidental or otherwise. If any question makes you too uncomfortable, you can skip it or stop the study.

You won't directly benefit from taking part in the study. This research will help us learn how to make period tracking apps better, especially for people your age.

If you don't want to be in the study, you don't have to participate. Remember, being in this study is up to you and no one will be upset if you don't want to participate or even if you change your mind later and want to stop.

If you provide an email address, you'll be entered into a drawing for a $100 gift card to Amazon or Starbucks (your choice).

You may contact the research team by phone (703-599-7653) or email (depstein@cs.washington.edu) if you have any questions about the study or if you feel you have been harmed by participating. If you would like to know more about your rights as a research participant or if you have a concern, please contact the Human Subjects Division at UW at (206) 543-0098.
