# Supplementary material for: Examining Menstrual Tracking to Inform the Design of Personal Informatics Tools
Source: Proc SIGCHI Conf Hum Factor Comput Syst. Author manuscript; Available in PMC 2017 May 15. (PMC5432133; doi:10.1145/3025453.3025635)
Supplement: 6 - Recruitment messages [file NIHMS855306-supplement-6_-_Recruitment_messages.pdf]

Tweet:

Record or predict when your period is happening? Help colleagues and I at UW understand how you keep track! [survey link]

Facebook post:

Do you keep a record of when your period has happened, or try to predict when your next period will be? We're running a study at the University of Washington to learn more about how people keep track of their periods. Please fill out our 15-minute survey! You'll be entered into a drawing for a $100 gift card. [survey link]

9. How do you keep track of your period? *

- ☐ I use a dedicated app on my phone/computer
- ☐ I use a calendar on my phone/computer
- ☐ I use a paper system, like a calendar or a diary
- ☐ I just remember
