# Supplementary material for: Examining Menstrual Tracking to Inform the Design of Personal Informatics Tools
Source: Proc SIGCHI Conf Hum Factor Comput Syst. Author manuscript; Available in PMC 2017 May 15. (PMC5432133; doi:10.1145/3025453.3025635)
Supplement: 7 - Methods details and codebooks [file NIHMS855306-supplement-7_-_Methods_details_and_codebooks.pdf]

Methods Details

App Reviews

We analyzed review data through a grounded approach. Two researchers open coded the reviews, generating nearly 30 codes before paring down to 6 codes most relevant to our research questions. Another then coded the entire corpus per these 6 codes, with two additional researchers coding 25% each (κ=0.66-0.80). One of the researchers who defined the codes broke any ties.

Survey

After analyzing the app reviews, we designed a survey to address open questions about why and how women track. To understand whether menstrual tracking practices differed in a generation who grew up with apps available or if practices differ based on experience with menstruation, we developed the survey to reach both teenagers (13-18) and adults (18+).

We obtained IRB approval for this study from our university as minimal risk research with a waiver of parental consent. Adults granted consent after reading a description of the study. Minors assented to participation after reading a similar description adjusted for a grade school reading level.

Requiring parental consent would impact ability to conduct the research and could increase the risk level of the study. Requiring minors to get approval from their parents could lead to an uncomfortable conversation or accidentally lead parents to make inferences about a minor's behavior, such as taking birth control without their permission or being sexually active. In addition, a signed parental consent form with a minor's name would be the only identifiable information in the study, potentially violating their anonymity.

Three researchers first read the open-ended survey responses and discussed potential codes. The first author open coded the responses before condensing to 14 codes most relevant to our research questions. Two researchers then coded 10% of the data. Code agreement varied on this initial pass (κ=0.31-1, with 0.80 or higher for 10 codes). The two researchers arbitrated the disagreements until reaching 100% agreement, and one researcher then coded the remainder of the data.

Interviews

Our analysis of survey responses revealed areas we wanted to explore in more detail, including situations where tracking was uncomfortable and the way in which menstrual tracking data is discussed with healthcare providers. We aimed for diversity in experiences and backgrounds (including race, gender, sexual minorities, and health conditions) rather than representativeness.

The researchers discussed major themes and identified 10 codes. Each transcript was then coded once by a researcher who did not participate in that interview, which helped each researcher become familiar with more interview data. We did not conduct inter-rater reliability on the interview data. It is rarely calculated on semi-structured interview data because people can apply the same code to different parts of a conversation. The interviews were conducted under the same minimal risk IRB as the survey.

Codebooks

App Reviews

We selected six codes most relevant to our research questions. Some codes had multiple levels.

1. accuracy (described app as accurate, not accurate)
2. sharing (doctor, partner, other family members, forum/community within the app, multiple)
3. app personality (discussed app's femininity, discreetness, or both as a positive or negative)
4. pregnancy (trying to conceive, trying to avoid)
5. life stages (teenager, pregnant, menopause)
6. quantified self practices

The other codes we developed in our open coding included features people wanted their apps to add (e.g., password protection, pregnancy mode), features people used frequently (e.g., glanceable displays, providing scientific reasoning), specifics of app personality (e.g., custom wallpapers, pink), and problems people had with app functionality (e.g., app would crash often, would lose their data).

Survey

The first author developed about 20 codes, which were condensed into 14:

- The 6 methods of tracking in Table 2 of the paper, plus "do not track"
- 4 categories of concern (aesthetics or features, gender or sexual identity, discreetness mitigated, discreetness unmitigated)
- 3 sources of recommendation for tracking method (friends, parents or family, doctors)

The other codes developed included more specific categories for concern (e.g., identifying as male, non-heteronormativity), other sources of recommendation (e.g., female partner, app store reviews), other ways people kept their apps discreet (e.g., password protecting, changing app names, using a foreign language).

Interviews

We had collected survey responses for the people we interviewed, so we did not code for again tracking method. We developed 10 codes:

- The same 4 categories of concern as the survey
- The same 3 sources of recommendation as the survey
- 3 more codes related to our interview motivations: thoughts on tracking sex and/or ovulation, other forms of tracking (e.g., activity tracking, financial tracking), and the change of tracking needs over time
